# Short Term Safety, Immunogenicity, and Reproductive Effects of Combined Vaccination With Anti-GnRH (Gonacon) and Rabies Vaccines in Female Feral Cats

**DOI:** 10.3389/fvets.2021.650291

**Published:** 2021-05-10

**Authors:** Shiri Novak, Boris Yakobson, Shir Sorek, Liat Morgan, Smadar Tal, Ran Nivy, Roni King, Lauren Jaebker, Douglas C. Eckery, Tal Raz

**Affiliations:** ^1^Koret School of Veterinary Medicine, The Robert H. Smith Faculty of Agricultural, Food, and Environment, The Hebrew University of Jerusalem, Rehovot, Israel; ^2^Kimron Veterinary Institute, Ministry of Agriculture, Rishon Lezion, Israel; ^3^Israel Nature and Parks Authority, Jerusalem, Israel; ^4^National Wildlife Research Center, United States Department of Agriculture Animal and Plant Health Inspection Service Wildlife Services, Fort Collins, CO, United States

**Keywords:** feral cats, anti-GnRH vaccine, Gonacon, non-surgical contraception, contraception, sterilization, rabies

## Abstract

Overpopulation of free-roaming cats is a major problem leading to negative impacts on animal health and welfare, public nuisance, transmission of zoonotic diseases, and well-documented harm to wildlife. Surgical sterilization had failed to provide a practical solution to free-roaming cats' overpopulation under field conditions; therefore, efficient and safe non-surgical immunocontraception methods are aspired. Rabies is a deadly virus that may infect people and animals. However, the safety and efficacy of combined vaccination with anti-GnRH and rabies vaccines in feral cats, which often suffer from disrupted health conditions and experienced high stress level, has never been studied. Therefore, our objective was to examine the short-term safety and efficacy of anti-GnRH vaccine (Gonacon), in combination with rabies vaccine in female feral cats. Mature feral female cats were captured and divided into the following groups: (I) GonaconX1-Rabies: queens vaccinated with both Gonacon and rabies (*n* = 5); (II) GonaconX2-Rabies: queens vaccinated twice with Gonacon (3 weeks apart) and with Rabies (*n* = 4); (III) OVx-Rabies: queens ovariohysterectomized and vaccinated with rabies (*n* = 4); (IV) Intact-Rabies: queens vaccinated against rabies and remained intact (*n* = 3). Comprehensive veterinary examinations and blood tests were performed every 2 weeks for 14 weeks. Data were analyzed by Repeated-Measures-ANOVA or Fisher-Exact-Test. There were neither systemic nor local adverse reactions at the vaccination sites. Blood count (PCV, TS, RBC, HGB, HCT, WBC) and chemistry (Total protein, Total globulin, Albumin, Urea, Creatinine, Creatine kinase, Bilirubin, GGT, ALT, AST) analyses revealed no differences among groups. There were no differences in serum rabies antibodies titers among groups, and queens kept a protective titer (>0.5 IU/mL) starting at 2–4 weeks after vaccination. Anti-GnRH antibodies were detected in all Gonacon-vaccinated queens, excluding one queen (GonaconX2-Rabies group). Anti-müllerian hormone serum concentrations reduced significantly after ovariohysterectomy, as well as gradually following vaccination with Gonacon, but it remained high in intact queens. Evaluation of vaginal cytology and ovarian histology suggested that reproductive cyclicity was suppressed in Gonacon-vaccinated queens. Our results support the conclusion that in the short term, the combined vaccination with Gonacon and rabies is safe and effective in female feral cats. However, further long-term studies are warranted to test this immunologic regimen in feral cats.

## Introduction

Overpopulation of free-roaming cats is a global problem in both urban and rural societies (1). It negatively impacts the cats' welfare and health (starvation, diseases, run over by cars, etc.), as well as causing public health risks due to the transmission of zoonotic diseases to humans (e.g., toxoplasmosis, rabies, cat scratch disease, leptospirosis, Q fever, toxocariasis, flea-borne typhus, etc.); some of these are reported to cause important health issues including abortion, blindness, pruritic skin rashes and other various symptoms, as well as mortality (2–6). Free-roaming abandoned and feral cats are non-native predators; they cause substantial wildlife destruction and ecosystem disruption, including the deaths of millions of birds, small mammals, reptiles, amphibians, and fish (7). Furthermore, disease transmission from cats to livestock and wild animals, attacks on humans, traffic accidents, and nuisance behavior, are just part of many concerns involved with this matter (6, 8, 9). Currently, surgical sterilization (ovariohysterectomy; orchiectomy) is the most common practice to control free-roaming cat reproduction, mainly *via* “Trap-Neutering-Return” (TNR) programs. It provides a permanent solution, should be done only once, and may positively affect the health and welfare of individual feral cats (10). However, the surgical approach is expensive, and requires surgical and anesthetic materials, equipment, as well as skilled veterinarians. Furthermore, it may cause significant stress to the animals (9, 11, 12). Therefore, the surgical approach practically failed to provide a solution for the millions of free-roaming cats that are confined and euthanized annually (9, 13–15). An efficient non-surgical sterilization/contraception method could have a significant impact on the control of cat overpopulation. However, to the best of our knowledge, despite significant efforts over the several last decades, there are currently no efficient commercial products that can be used for mass non-surgical sterilization/contraception of free-roaming cat populations (15, 16).

Rabies is a deadly, vaccine-preventable, zoonotic, viral disease, which may infect people and animals (17). According to the World Health Organization report (updated in April 2020), most human cases worldwide are due to disease transmission from domestic dogs. However, mandatory dog vaccination programs have halted the natural spread of rabies among domestic dogs in many countries. According to the information from the USA Centers for Disease Control and Prevention (CDC; updated on April 2020), as well as from other reports (4, 18, 19), in the USA, cats have become the companion animal species most commonly reported as rabid. Accordingly, in many endemic countries, including Israel and the USA, combined animal reproduction control and rabies vaccination programs are aspired.

Reproduction in the domestic queens is affected by photoperiod, as this species is considered as seasonal, polyestrous, long day breeder, with multiple ovulations induced following vaginal stimulation during mating (20, 21). Gonadotropin-Releasing Hormone (GnRH), which is released from the hypothalamus, is the master hormone of reproduction; it controls steroidogenesis and gametogenesis by stimulating the release of the pituitary gonadotropins, luteinizing hormone (LH) and follicle-stimulating hormone (FSH), triggering a cascade of endocrine effects that lead to sperm production in males and follicular development and ovulation in females. Gonacon is an anti-GnRH vaccine, developed by the National Wildlife Research Center, USDA APHIS Wildlife Services, USA. This immunocontraceptive vaccine was designed to trigger the production of antibodies that neutralize GnRH. It has been tested in many species, including white-tail deer (22), elk (23), horses (24, 25), cattle (26), ferrets (27), domestic, and wild pigs (28), as well as to a limited extent in dogs (29) and cats (30–33). However, none of the four previous studies conducted on cats included measurements of rabies antibodies in Gonacon-vaccinated cats, even in the studies in which cats were vaccinated with both vaccines (32, 33). Furthermore, in previous Gonacon studies conducted on cats, animals were not feral cats; they were either specific-pathogen-free (SPF) laboratory cats (30, 31), cats from research colony (32), or well-maintained friendly cats obtained from animal control agencies or from private individuals who posted cats for rehoming (33). All cats were kept in excellent health and body conditions, and were accustomed to human interactions. However, many previous reports documented that feral cats often suffer from high rates of morbidity and pre-mature mortality due to infectious (e.g., FIV, FeLV, parasites) and non-infectious diseases, malnutrition, poor body condition, traumas, etc. (9, 10, 34–36). Furthermore, most of these cats are typically too fearful and too wild to be handled awake, as they are not accustomed to close interactions with people. Therefore, feral cats, which are the main target population for non-surgical contraception/sterilization, often suffer from acute and chronic health problems and are under chronic stress due to their lifestyle and environment (5, 9, 10, 34–36). Potentially, these suboptimal conditions might weaken the immune response and antibody production following vaccination, such as to Gonacon and rabies vaccines, as has been documented for other vaccines and species (37–42). As recently reviewed by Zimmermann and Curtis (41), several factor might affect the immune respond and antibody production of individuals following vaccination; these include age, sex, genetics, comorbidities, infections, parasites, microbiota, pre-existing immunity, acute and choric stress, body condition, nutritional status, micronutrients (vitamins A, D, and E and zinc), as well as environmental factors, and vaccine factors (vaccine type, product, strain, and batch; vaccination route; combinations of vaccines, cet.). For example, some studies indicated poorer humoral response to influenza vaccine in humans with chronic diseases (37); Chronic inflammatory processes like rheumatoid arthritis alter the immune system and antibody formation (43); furthermore, depression and dementia as well as psychological stress are associated with poor antibody response and a higher range of inflammation markers (44). Thus, vaccines should optimally be tested in the typical target population and conditions.

Therefore, the objective of the current study was to examine the short-term safety and efficacy of the anti-GnRH vaccine (Gonacon), given either as a single dose, or two doses 3 weeks apart, in combination with rabies vaccine, in mature feral female cats. Our main hypotheses were that: (1) in the short term (14 weeks), the combined vaccination with Gonacon and rabies will not be harmful and will not have adverse side effects; (2) antibody titers against GnRH and rabies will increase following the combined vaccination; (3) as compared to a single dose Gonacon vaccination, two doses of Gonacon given 3 weeks apart (booster) will induce higher anti-GnRH titer, but anti-rabies antibodies titer will not differ; (4) development of anti-GnRH antibodies will be associated with suppression of the ovarian function; and (5) antibody titers against rabies will not be negatively influenced by the simultaneous vaccination approach and will remain protective during the study period.

## Materials and Methods

### Cats, Housing, and Handling

The study was conducted according to the ethical approval of the Hebrew University's Institutional Animal Care and Use Committee (IACUC MD-17-14613-2). As part of the trap-neuter-return (TNR) program in a large city in Israel (Be'er-Sheva), free-roaming feral domestic short-hair (DSH) queens were captured by professional trappers using commercial traps, and were transported to the cats' housing facility located at the municipal veterinary department. Mature intact queens (*n* = 16; estimated age ranged from 1 to 3 years), with no severe injuries, were randomly selected for the study, which was conducted during the natural breeding season of cats in Israel (July to September). During the 14-weeks study, queens were housed individually in cages in the same ventilated room and maintained at ambient temperature and light. They could see and smell each other, without direct contact. Food and water were available *ad libitum*, and environmental enrichment was provided in each cage.

Queens were observed by professional veterinarians and staff, at least twice every day during the 14-weeks study period. The attitude, appetite, and alertness of the queens were evaluated while the cats were in their cages. However, these queens could not be handled safely while awake, as they were not friendly and not used to any human contact (typical for most free-roaming cats in Israel). Therefore, full physical examinations, treatments, and sampling (blood and vaginal smears) were performed under heavy sedation. Every 2 weeks, starting at the vaccination day, the queens were sedated using a combination of Medetomidine (0.02–0.04 mg/kg; Orion Pharma, Finland), Butorphanol (0.2–0.4 mg/kg; intramuscular injection; Richter Pharma AG, Austria), and Midazolam (0.1–0.25 mg/kg intramuscular injection; Rafa laboratories, Israel). At the end of the procedure, Medetomidine was reversed by subcutaneous administration of atipamezole (0.05–0.10 mg/kg; Orion Pharma, Finland). After the study was completed, cats that had relatively calm temperaments were acclimated, and thereafter were adopted. Others were released back to the exact location in which they were initially trapped, in compliance with the Israeli Feral Cat Low. All queens were in good health and body condition at the time of adoption or release.

### Treatments and Study Design

At study initiation, queens were allocated into one of the following groups: (I) GonaconX1-Rabies: queens were vaccinated with both Gonacon and rabies vaccines (*n* = 5); (II) GonaconX2-Rabies: queens were vaccinated twice with Gonacon (at study initiation, and again 3 weeks later) and once with rabies vaccine, at study initiation (*n* = 4); (III) OVx-Rabies: queens underwent surgical ovariohysterectomy and vaccinated against rabies at study initiation (*n* = 4); (IV) Intact-Rabies: queens vaccinated against rabies and remained intact (*n* = 3). Vaccination against rabies was performed at study initiation in all queens by subcutaneous administration of a commercial rabies vaccine (Rabisin, Merial, France) at the left thigh. Vaccination with Gonacon was performed by intramuscular administration of vaccines provided by USDA-APHIS NWRC (Fort Collins, CO, USA). The Gonacon vaccine was injected into the right quadriceps muscle group. Before the administration of the vaccines, the fur was clipped, and the injection sites were cleaned with 70% isopropyl alcohol.

### Monitoring and Sampling

Throughout the study, queens were monitored daily for their attitude, appetite, and alertness. Every 2 weeks, for a total period of 14 weeks, full physical examinations were performed by a certified veterinarian, including also evaluation of body condition on a 5-point scale (45), examinations of the injection sites, as well as collections of vaginal smears. Furthermore, blood samples were collected by jugular venipuncture every 2 weeks into potassium-EDTA tubes, as well as into plain tubes containing no anticoagulant. Blood samples in potassium-EDTA tubes were stored at 4°C and used for complete blood count (CBC) analysis within 4 h. Blood in plain tubes was allowed to clot, centrifuged, and serum samples were stored at −80°C until analyses at study completion, which included serum chemistry, anti-GnRH and anti-rabies antibody titer, serology for FIV and FeLV, as well as analysis of serum Anti Müllerian Hormone (AMH) concentration. At study termination, all queens underwent surgical ovariohysterectomy, except the cats in the OVx-Rabies group, in which ovariohysterectomy was performed at study initiation. The removed reproductive tracts from all queens were kept and processed for histological evaluation, as detailed below.

### Complete Blood Count and Blood Chemistry Analyses

Blood samples collected in potassium-EDTA tubes on weeks 0, 2, 4, 6, 8, 10, 12, and 14 were used for complete blood count (CBC; Advia 2120, Siemens, Erfurt, Germany). Packed cell volume (PCV) was measured routinely, using a hematocrit tube sealed with clay that was centrifuged for ~3 min; plasma total solids was measured by placing the plasma directly onto a refractometer (Clinical Refractometer RHC-200 ATC, Cmall, Medent, Israel). Blood smears were prepared and stained by automatic slide Stainer with a modified Wright's staining solution (Modified Wright's Stain, Bayer Hematek 2000 Slide Stainer, Siemens, Elkhart, IN, USA), and used for microscopic evaluation of blood cells. Neutrophil cytoplasmic toxicity was evaluated microscopically as previously described (46), by an experienced observer who was blinded to the identity of the queens and treatments (Dr. Nivi, DVM, Dip.ECVIM-CA).

Serum samples collected on weeks 0, 2, 8, and 14 were used for chemistry analysis (Cobas Integra 400 Plus; Roche, Mannheim, Germany, at 37°C). The analysis included the following parameters: Urea, Creatinine, Albumin, Alanine Amino-Transferase (ALT), Aspartate Amino-Transferase (AST), Gamma-Glutamyl Transpeptidase (GGT), Bilirubin, Creatine Kinase (CK), Globulins, and Total protein (TP).

### Detection of GnRH Antibodies

Serum samples were sent frozen to USDA-APHIS NWRC and were analyzed for GnRH antibodies using an Enzyme-Linked Immunosorbent Assay, as previously described (31). Serum samples were diluted in two-fold series from 1:8,000 to 1:64,000 in phosphate-buffered saline (PBS) and run in duplicate. A positive control sample was also run in duplicate on each plate. The cutoff values for each plate were generated from the pre-vaccination samples from all animals at each dilution as the mean + 3SD. The %CV for the positive control samples at each dilution was low, demonstrating little inter-plate variability (1:8,000, 7.3%; 1:16,000, 5.9%; 1:32,000, 8.2%, 1:64,000, 11.2%).

### Detection of Anti-Rabies Antibodies

Serum samples collected on weeks 0, 2, 4, 6, 8, 10, 12, and 14 were analyzed for anti-Rabies antibodies concentrations using Rapid Fluorescent Focus Inhibition Test (RFFIT), as previously described (47), in the OIE-accredited laboratory of Dr. Boris Yakobson at the Kimron Veterinary Institute (Veterinary Services and Animal Health, Israel). The RFFIT assay has been shown to have a sensitivity and specificity of 100 and 89%, respectively, and is considered as the gold standard by the World Health Organization (47, 48). Briefly, titers were recorded as serial dilutions with positive and negative control samples. All serum dilutions were added with an equal volume of virus and incubated at 37°C in a controlled humidity carbon dioxide 0.5% chamber for 90 min. Then, a suspension of 1 × 10^5^ cells (MNA-Mouse neuroblastoma, CDC USA, Wistar Institute, Philadelphia, USA) in 0.2 mL of growth medium (MEM EAGLE, with fetal bovine serum, vitamin solution concentrated, L-Glutamine, Sodium Bicarbonate, Penicillin G, Streptomycin and Amphotericin B; Biological Industries, Israel) was added to each well, and the chamber was returned to incubation for 20 h. Thereafter, the growth medium was removed, the chambers were rinsed in phosphate-buffered saline (PBS), and the cells were fixed with cold acetone (−20°C) at room temperature for 10 min. FITC anti-rabies conjugate (Fujirebio, USA) was added, and the chamber was incubated for 30 min in a humidity incubator (37°C), and finally washed with PBS. The slides were examined with a microscope (Olympus BX40, 470 excitation wavelength, ×200 magnification). Each well was divided into 20 fields, and the number of fields containing fluorescent cells was tabulated (49). Serological titers were converted to international units per mL (IU/mL). A titer value of >0.5 IU/mL was considered as a protective antibody level (47).

### Serum Anti Müllerian Hormone Analysis

Serum anti-Müllerian hormone (AMH) concentrations were analyzed in samples from weeks 0, 8, and 14 using an Enzyme-Linked Immunosorbent Assay kit (AMH Gen II ELISA; Beckman coulter, Inc. Brea, CA, USA) according to the manufacturer's instructions and as previously described and validated for cats (50). Briefly, 300 μL of AMH Gen II Assay Buffer were mixed with 60 μL of calibrator, control, or sample, and 120 μL from the mix were pipetted into the wells in the plate, in duplicates. After incubation and washing, 100 μL of anti-AMH biotin conjugate was added to each well. After a second incubation and washing, 100 μL of the streptavidin-enzyme conjugate were added. Following a third incubation and washing step, 100 μL of TMB chromogen solution were added, followed by the final addition of 100 μL of an acidic stopping solution. The degree of enzymatic turnover of the substrate was determined by using a microplate reader (SpectraMax Paradigm Multi-mode detection platform, Molecular Devices, Austria) using dual-wavelength absorbance measurement at 450 and 620 nm. The inter- and intra- coefficient of variations (%CV) were 5.4 and 4.2%, respectively.

### Vaginal Cytology

A fine cotton swab was used to obtain cells from the cranial vagina, which were spread on glass slides, as previously described (51). Slides were stained with Diff-Quick, and were evaluated microscopically (1,000 × magnification. Primo Star, Carl Zeiss, Germany) by a single experienced observer, who was blinded to the origin of the slides and the status of the queens. For each Diff-Quick stained slide, >200 epithelial vaginal cells were classified as parabasal, intermediate or superficial (nucleate and anucleate) cells (52). If >60% of the vaginal cells were superficial cells, the queen was considered to be in proestrus/estrus.

### Ovarian and Uterine Histology

Ovaries and uterine tissues collected during ovariohysterectomies were fixed immediately after collection in Bouin's solution for 24 h at 4°C, and then were moved to 70% ethanol. Tissues were embedded in paraffin, sectioned (4 μm thickness), and stained with hematoxylin and eosin (H&E; Sigma-Aldrich, Israel). Histological evaluation of these sections was performed using a light microscope (Axio Imager M1, AxioCam HRc camera; Carl Zeiss, Germany). In the ovary, we subjectively assessed the general morphology and presence of primordial follicles, antral follicles, corpora lutea, and oocytes; in the uterus, we assessed the general structural layers, as well as the competence of the luminal epithelium, and morphology of the endometrial glands within the endometrium.

### FIV/FeLV Serology

As part of the evaluation of the cats' health, serum samples collected prior to vaccination (week 0) were analyzed to detect antibodies for Feline immunodeficiency virus (FIV) and Feline leukemia virus (FeLV), using FIV/FeLV antibody commercial test kit (SNAP^*^ Combo plus, IDEXX, USA), according to the manufacturer's instructions.

### Statistical Analysis

Statistical analyses were performed using Statistix 8 software (Analytical Software, Tallahassee, FL USA); plots were produced by Prism 5.01 (GraphPad Software; San-Diego, CA, USA). Continuous data were analyzed by Repeated-Measures-ANOVA, to evaluate the effects of Group (between-subject factor), Time (within-subject factor), and Group-by-Time interactions, followed by Tukey HSD All-Pairwise Comparisons Test. When was applicable, two groups were compared by Wilcoxon Rank Sum Test. Fisher-Exact-Test was used to compare proportional data. Differences were considered significant at *P* < 0.05. Unless otherwise noted, results are presented as mean ± SEM.

## Results

### Physical Examinations, Complete Blood Count, and Blood Chemistry Analyses

**Figure 1:**
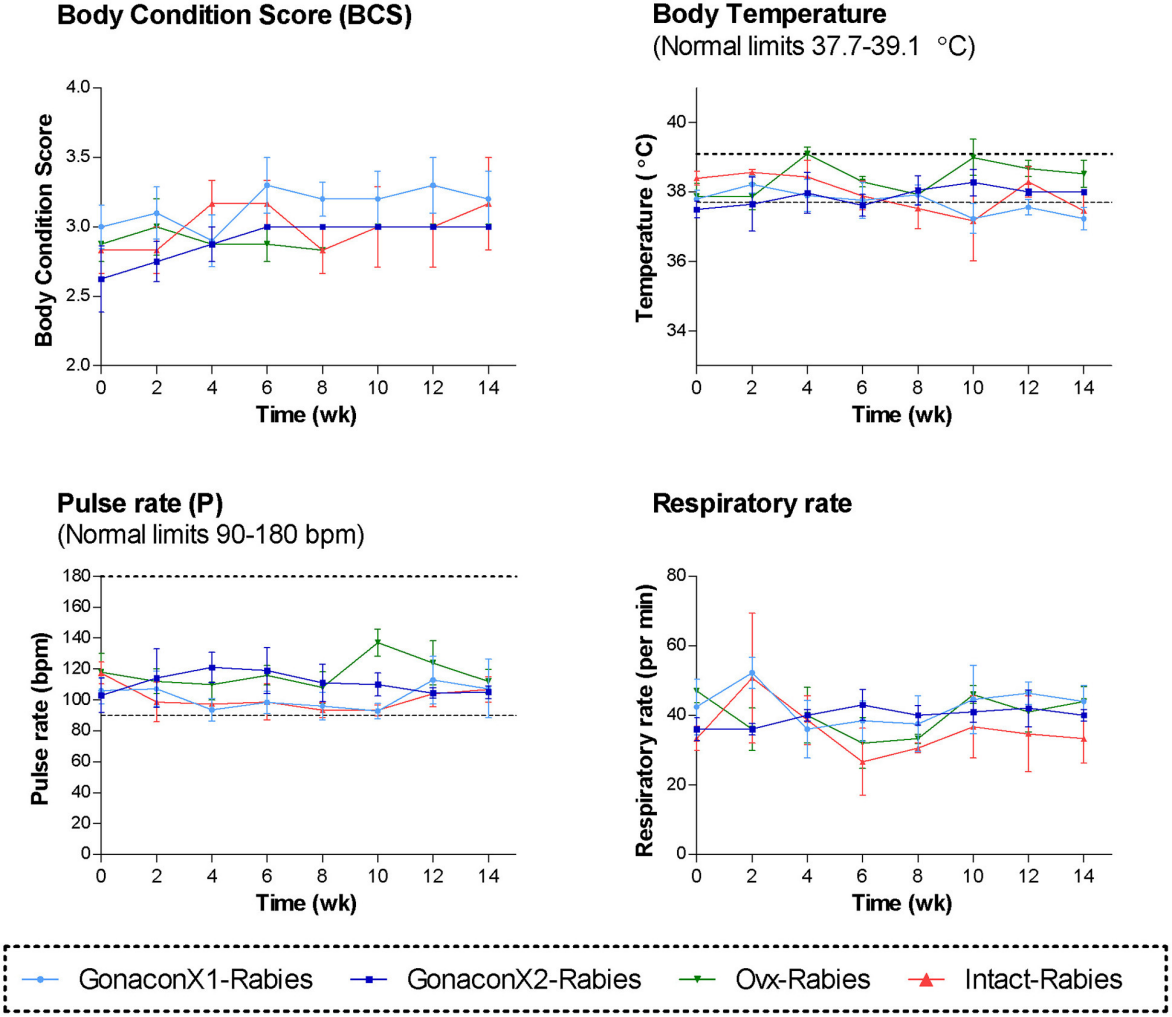
Body condition score and the vital signs of cats in the research groups. Mean ± SEM of temperature, pulse, respiratory rate, and body condition score are presented for each group: GonaconX1-Rabies: cats vaccinated with both Gonacon and Rabies (*n* = 5); GonaconX2-Rabies: cats vaccinated twice with Gonacon (3 weeks apart) and with rabies (*n* = 4); OVx-Rabies: cats ovariohysterectomized and vaccinated with rabies (*n* = 4); Intact-Rabies: cats vaccinated against rabies and remained intact (*n* = 3). Parameters were collected every 2 weeks for a total of 14 weeks, while cats were under heavy sedation. Dotted lines indicate normal limits.

During the course of the study, no swelling, signs of inflammation, or tenderness were detected at the injection sites, in any of the queens. All queens were in fair to good physical condition, and had a normal attitude and appetite. Vital signs (TPR; Temperature, Pulse, and Respiratory rates) remained within normal ranges, with no significant differences among the groups or over time (Figure 1). Body condition score (BCS) did not differ among the groups, but overall, it improved over the study period (*p* = 0.0281); BCS of individual cats stayed the same or increased by up to one point in a 5-point BCS scale (45).

**Figure 2:**
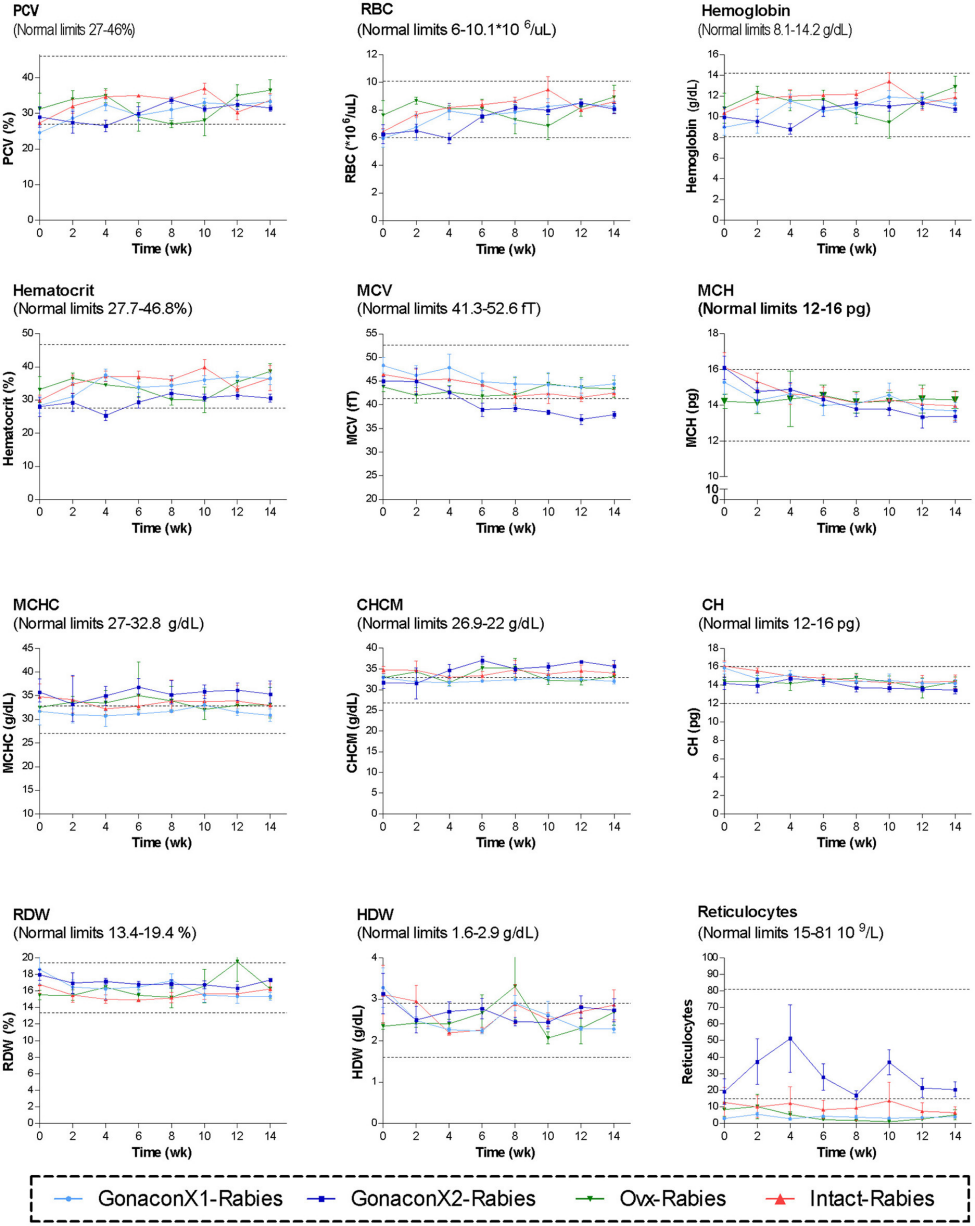
Red blood cells parameters of cats in the research groups. Mean ± SEM of red blood cells parameters are presented for each group: GonaconX1-Rabies: cats vaccinated with both Gonacon and rabies (*n* = 5); GonaconX2-Rabies: cats vaccinated twice with Gonacon (3 weeks apart) and with rabies (*n* = 4); OVx-Rabies: cats ovariohysterectomized and vaccinated with rabies (*n* = 4); Intact-Rabies: cats vaccinated against rabies and remained intact (*n* = 3). Blood samples were collected every 2 weeks for a total of 14 weeks. Dotted lines indicate normal limits. PCV, packed cell volume; RBC, red blood cell count; MCV, mean corpuscular hemoglobin; MCH, mean corpuscular hemoglobin concentration; MCHC, mean corpuscular hemoglobin concentration; CHCM, cell hemoglobin concentration mean; CH, cellular hemoglobin; RDW, red cell distribution width; HDW, hemoglobin distribution width.

**Figure 3:**
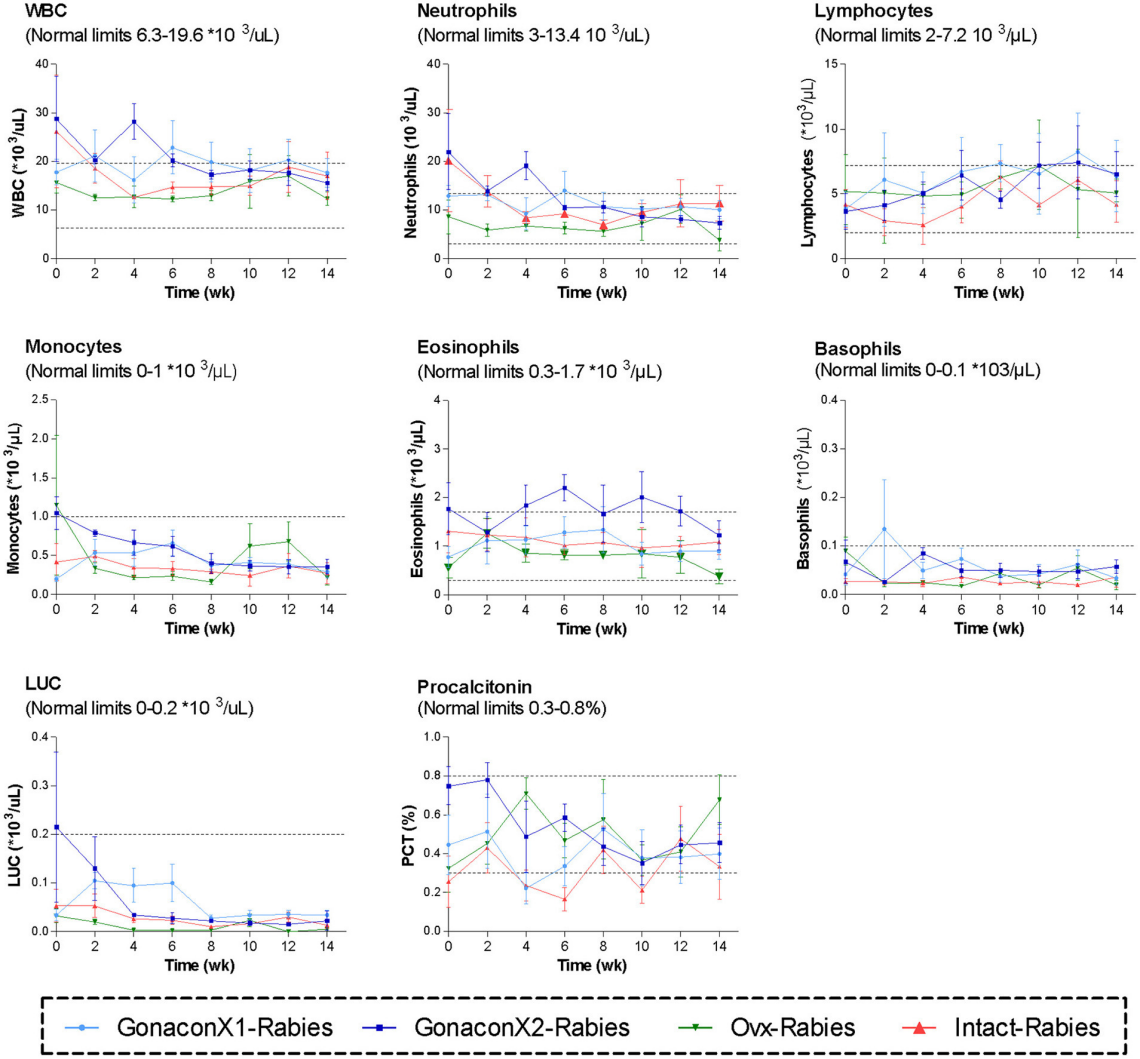
White blood cells parameters of cats in the research groups. Mean ± SEM of white blood cells parameters are presented for each group: GonaconX1-Rabies: cats vaccinated with both Gonacon and rabies (*n* = 5); GonaconX2-Rabies: cats vaccinated twice with Gonacon (3 weeks apart) and with rabies (*n* = 4); OVx-Rabies: cats ovariohysterectomized and vaccinated with rabies (*n* = 4); Intact-Rabies: cats vaccinated against rabies and remained intact (*n* = 3). Blood samples were collected every 2 weeks for a total of 14 weeks. Dotted lines indicate normal limits. WBC, white blood cells count; LUC, large unstained cells.

Complete blood count (CBC) was completed for all the samples (8 samples for each cat). There were no significant differences among the groups in all the CBC parameters (Figures 2, 3). However, in some individuals, some of the parameters were abnormal at study initiation, mainly indicating mild anemia or inflammatory condition, but they improved over the study period, and were within normal limits later on. Accordingly, there were significant differences along time (Repeated Measure ANOVA; *p* < 0.05) in the following parameters; PCV, TS, RBC, Hemoglobin, HCT, MCH, MCV, HDW, CH, WBC, Neutrophils, Lymphocytes, and Large unstained cells (LUC, which are activated lymphocytes and peroxidase-negative cells). In addition, overall, signs of neutrophil toxicity were common at study initiation, but were absent at study completion (6/16, 38% vs. 0/16, 0%; *p* = 0.0177).

**Figure 4:**
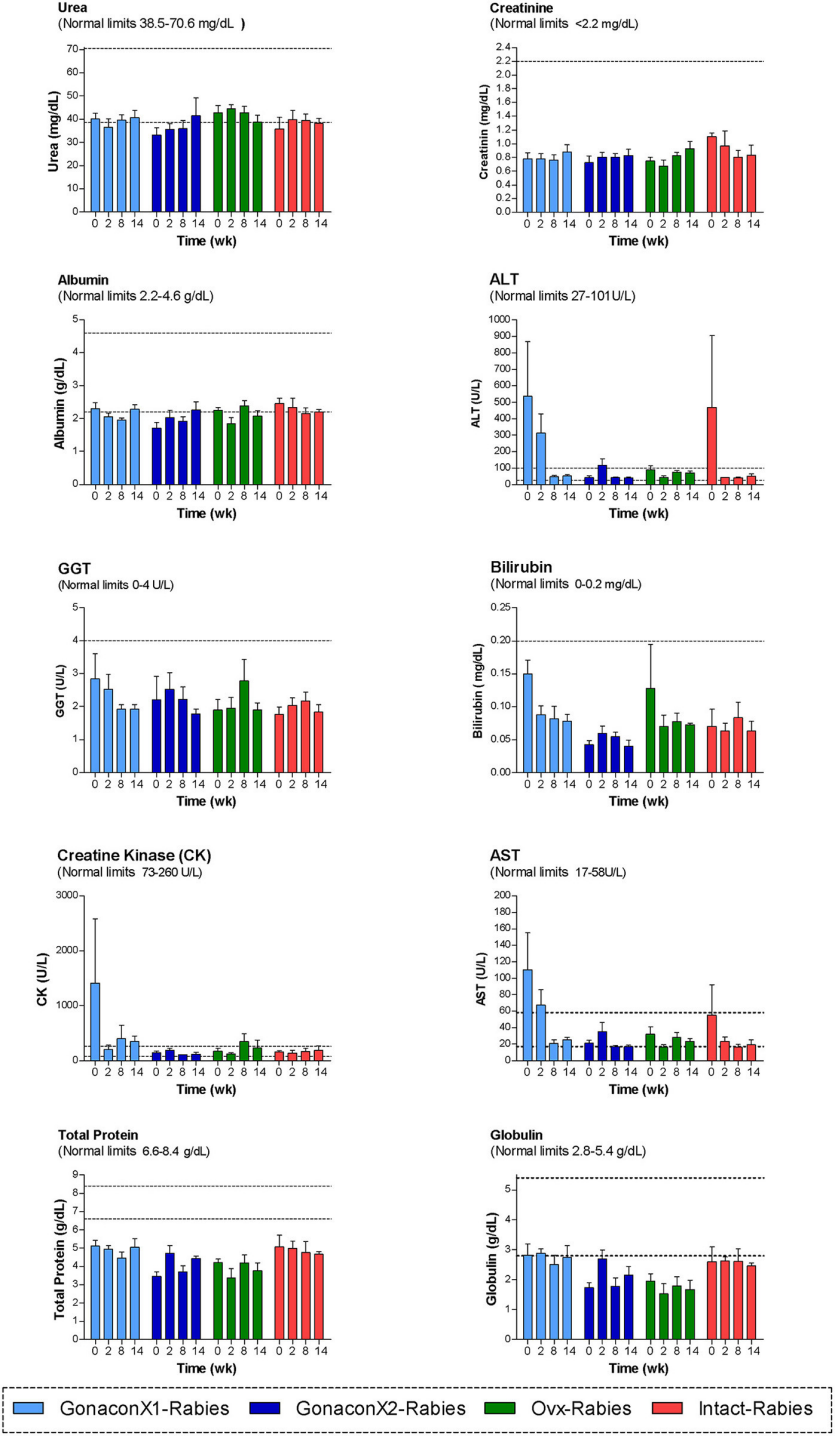
Blood chemistry parameters of cats in the research groups. Mean ± SEM of blood chemistry parameters are presented for each group: GonaconX1-Rabies: cats vaccinated with both Gonacon and rabies (*n* = 5); GonaconX2-Rabies: cats vaccinated twice with Gonacon (3 weeks apart) and with rabies (*n* = 4); OVx-Rabies: cats ovariohysterectomized and vaccinated with rabies (*n* = 4); Intact-Rabies: cats vaccinated against rabies and remained intact (*n* = 3). Analyses were performed on blood samples collected at 0, 2, 8, and 14 weeks after the initial vaccination. Dotted lines indicate normal limits. ALT, alanine aminotransferase; GGT, gamma-glutamyl transpeptidase; AST, aspartate aminotransferase.

Analyses of blood chemistry parameters were conducted on blood samples from weeks 0, 2, 8, and 14, to overall estimate the function of internal organs, such as liver and kidney, after vaccination. As illustrated in Figure 4, there were no significant differences among the groups at the chemistry parameters, except for a few parameters, at study initiation, before the queens were vaccinated; mean AST and CK concentrations were significantly high at the GonaconX1 group, and ALT was high in both GonaconX1-Rabies and Intact-Rabies groups (*p* < 0.05). However, all parameters improved over the study period, and were within normal limits later on, excluding albumin and total proteins, which were relatively low throughout the study.

None of the queens in the study had positive serology for FeLV; however, one queen in the GonaconX1-Rabies group was positive for FIV.

### GnRH Antibodies

**Figure 5:**
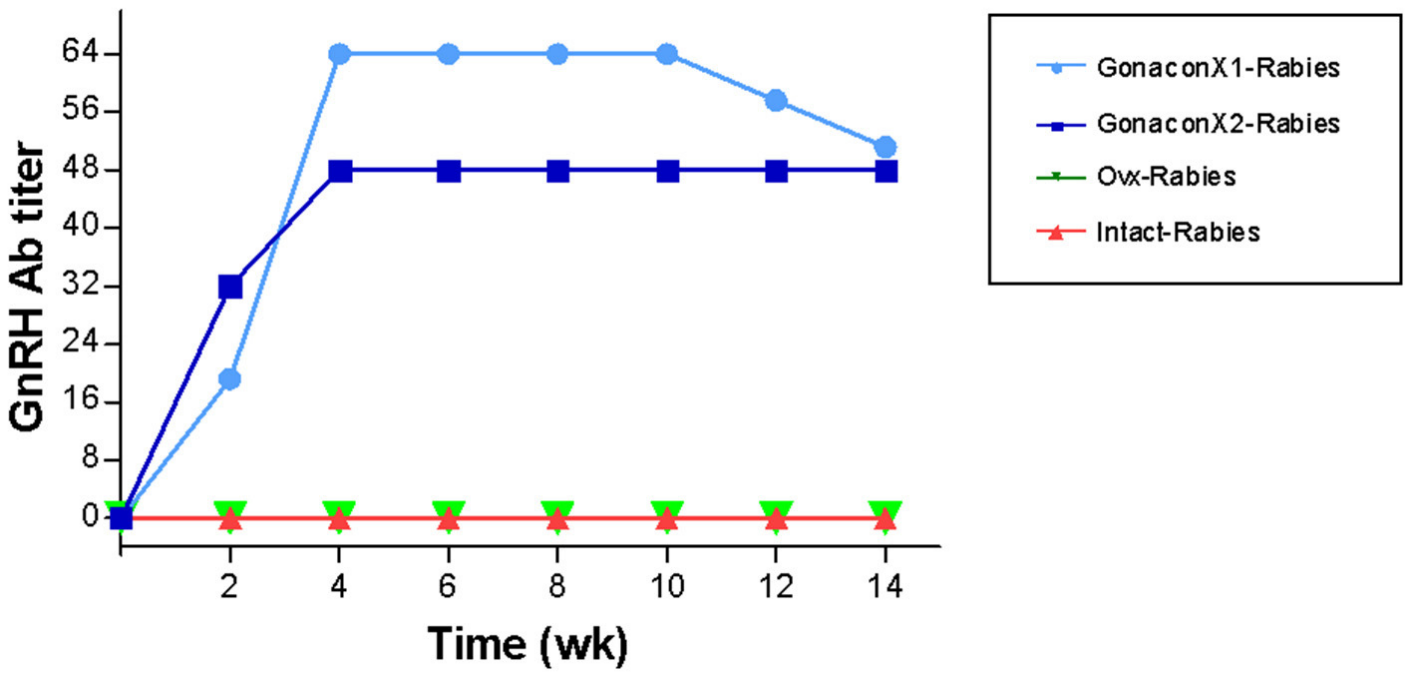
Serum GnRH antibodies titer of cats in the research groups. Mean ± SEM GnRH titers are presented for each group: GonaconX1-Rabies: cats vaccinated with both Gonacon and rabies (*n* = 5); GonaconX2-Rabies: cats vaccinated twice with Gonacon (3 weeks apart) and with rabies (*n* = 4); OVx-Rabies: cats ovariohysterectomized and vaccinated with rabies (*n* = 4); Intact-Rabies: cats vaccinated against rabies and remained intact (*n* = 3). Blood samples were collected every 2 weeks for a total of 14 weeks. There were significant effects of the group, the time, and the group X time interaction on the anti-GnRH antibodies titer (Repeated measure ANOVA, *p* ≤ 0.0003). Variability within each group was extremely small, and therefore the SEM are unnoticeable.

**Figure 6:**
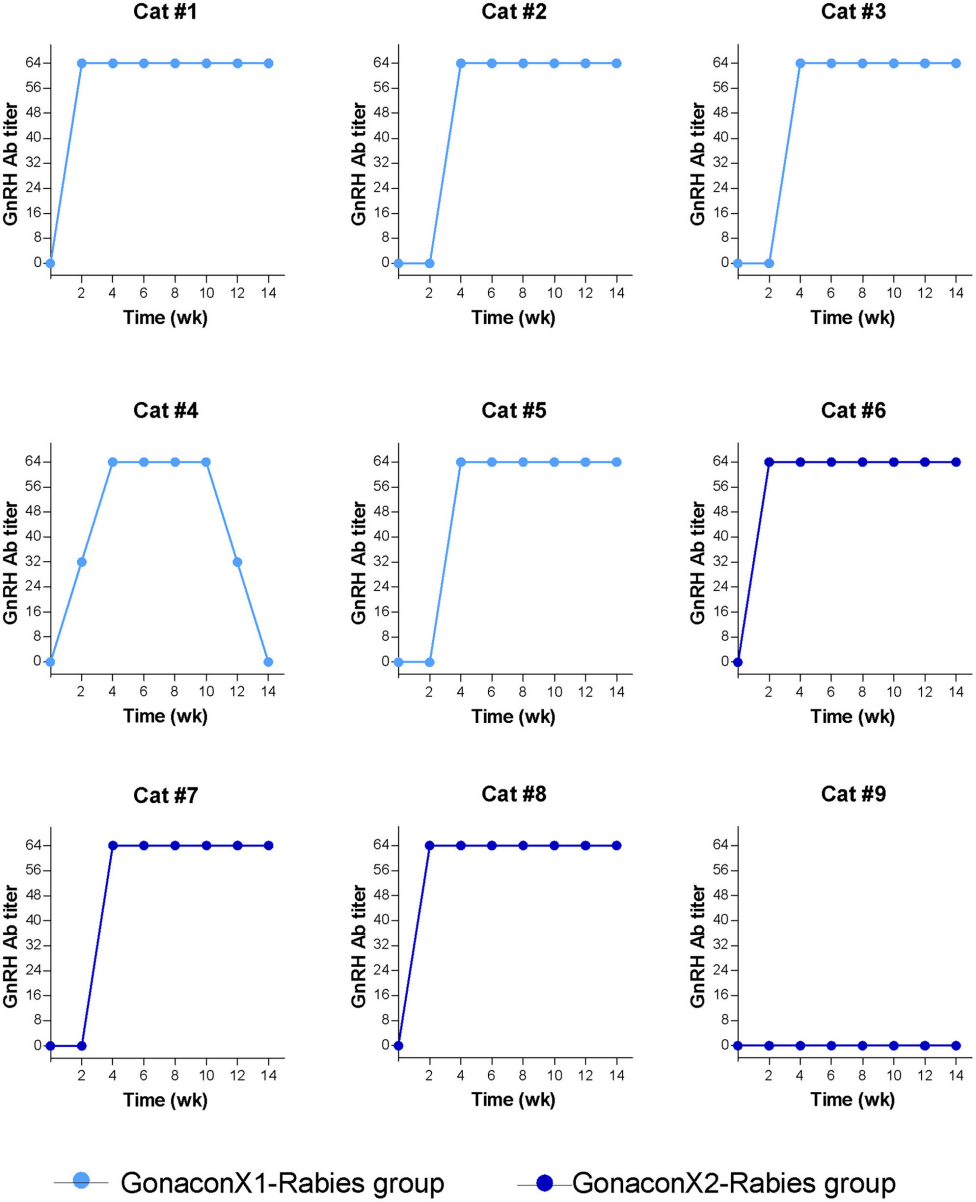
Serum GnRH antibodies titer of individual cats vaccinated with Gonacon. Each graph represents data of a single cat. GonaconX1-Rabies group: cats vaccinated once with both Gonacon and rabies (*n* = 5; light blue); GonaconX2-Rabies group: cats vaccinated twice with Gonacon (3 weeks apart) and once with rabies (*n* = 4; dark blue); Blood samples were collected every 2 weeks for a total of 14 weeks. Note the reduction of antibodies in cat#4 at weeks 12 and 14, as well as the non-responder cats (#9) in the GonaconX2-Rabies group.

There were significant effects of the group, the time, and the group X time interaction on the anti-GnRH antibody titer (*p* ≤ 0.0003; Figure 5). As expected, anti-GnRH antibodies were not detected in any the control queens (OVx-Rabies and Intact-Rabies groups). However, following vaccination with Gonacon, anti-GnRH antibodies titer increased in all vaccinated queens (GonaconX1-Rabies and GonaconX2-Rabies groups), except for one queen (cat #9) from the GonaconX2-Rabies group, in which no anti-GnRH antibodies could not be detected in any of its serum samples (Figure 6). There was no significant difference between the GonaconX1-Rabies and GonaconX2-Rabies groups (*p* = 0.4943). In all responder queens, positive anti-GnRH antibody titers were detected, typically within 2 or 4 weeks, and remained high during the study period; except for one queen from the GonaconX1-Rabies group (cat #4), in which antibodies decreased in the last two samples. This queen was identified later on as FIV positive.

### Rabies Antibodies

**Figure 7:**
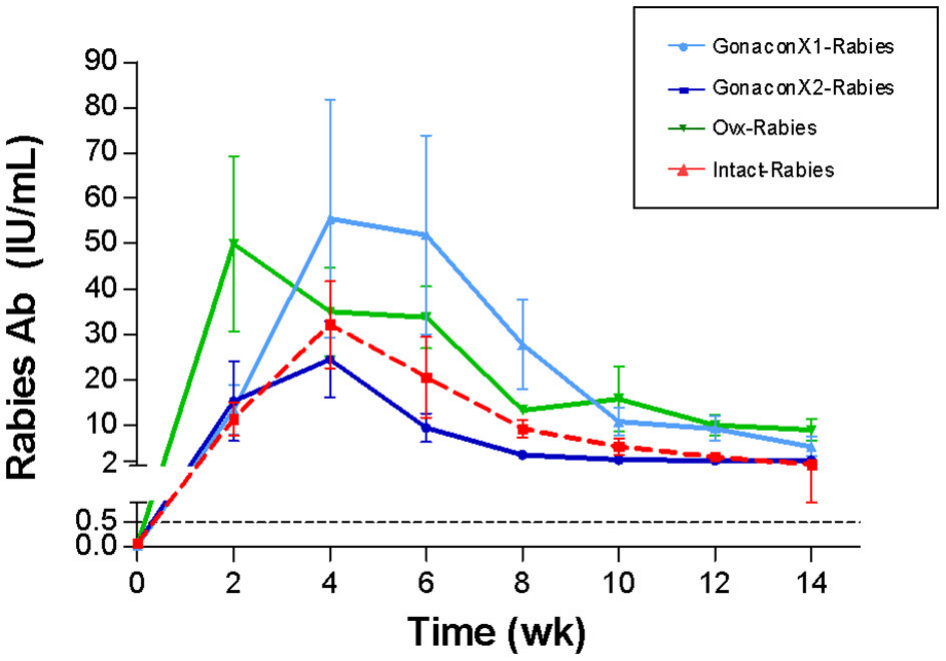
Serum rabies antibodies of cats in the research groups. Mean ± SEM rabies antibodies concentrations are presented for each group: GonaconX1-Rabies: cats vaccinated with both Gonacon and rabies (*n* = 5); GonaconX2-Rabies: cats vaccinated twice with Gonacon (3 weeks apart) and with rabies (*n* = 4); OVx-Rabies: cats ovariohysterectomized and vaccinated with rabies (*n* = 4); Intact-Rabies: cats vaccinated against rabies and remained intact (*n* = 3). Blood samples were collected every 2 weeks for a total of 14 weeks. There were no significant differences in the rabies antibodies among the groups (*p* = 0.3169), or group X time interaction (*p* = 0.7220); however, the time was a significant factor (Repeated measure ANOVA, *p* < 0.0001). Dotted lines indicate protective titer (>0.5 IU/mL).

There were no significant differences in the anti-rabies antibodies among groups (*p* = 0.3169), or group X time interaction (*p* = 0.7220); however, time was a significant factor (*p* < 0.0001; Figure 7). Following vaccination with rabies, all queens from all the groups produced antibodies against rabies, which remained above 0.5 IU/mL throughout the study, a titer that is considered protective. Typically, the anti-rabies antibodies titer peaked at 4–8 weeks following vaccination, and then gradually reduced and appeared to be stabilized.

### Anti-müllerian Hormone Serum Concentrations

**Figure 8:**
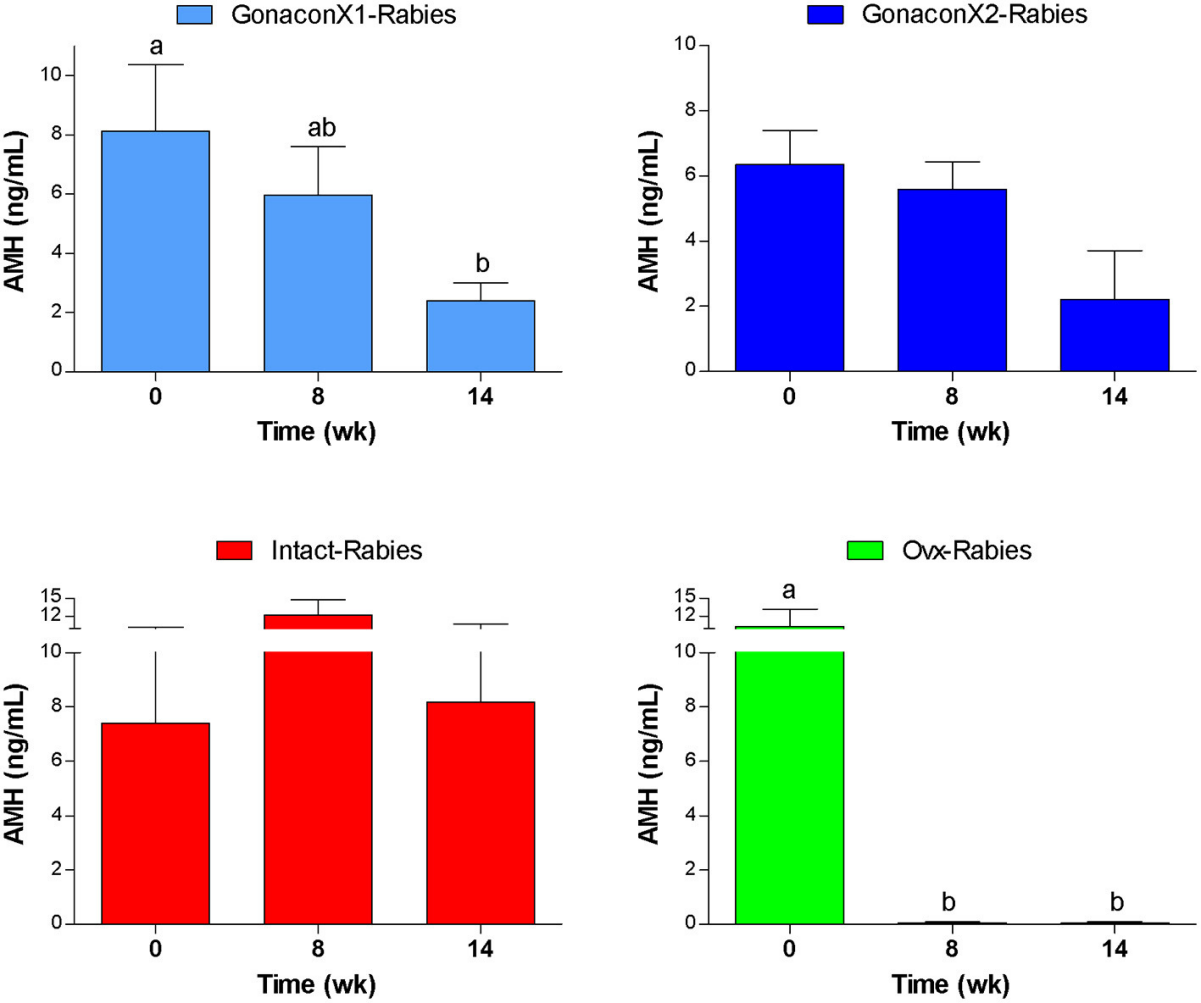
Serum concentrations of anti-müllerian hormone of cats in the research groups. Serum concentrations of anti-müllerian hormone (Mean ± SEM) are presented for each group: GonaconX1-Rabies: cats vaccinated with both Gonacon and rabies (*n* = 5); GonaconX2-Rabies: cats vaccinated twice with Gonacon (3 weeks apart) and with rabies (*n* = 4); OVx-Rabies: cats ovariohysterectomized and vaccinated with rabies (*n* = 4); Intact-Rabies: cats vaccinated against rabies and remained intact (*n* = 3). Analyses were performed on blood samples collected at 0, 8, and 14 weeks after the initial vaccination. ^a, b^Different letters above bars represent significant differences (*p* < 0.0.5).

As illustrated in Figure 8, AMH serum concentrations reduced significantly after ovariohysterectomy (*p* = 0.0023), but it remained high in intact queens (*p* = 0.4174). In queens vaccinated with Gonacon, the reduction in AMH concentration was gradual (*p* = 0.0006). In all Gonacon-vaccinated queens, AMH serum concentrations were lower at study completion, as compared to study initiation, except in the non-responder queen in the GonaconX2-Rabies, who kept relatively stable AMH concentrations. In the GonaconX1-Rabies group, mean AMH concentration was significantly lower at study completion, as compared to study initiation (*p* = 0.0081). In the GonaconX2-Rabies group, mean AMH concentration was not statistically different at study completion (*p* = 0.1257); however, when the data of the non-responder queen was excluded from the analysis, the difference in AMH concentrations between study initiation and study completion was significant also in that group (*p* = 0.0178).

### Vaginal Cytology

The analysis of the percentage of superficial cells in vaginal cytology smears by Repeated Measure ANOVA revealed no significant effect of the group (*p* = 0.6286), time (*p* = 0.4027), and group X time interaction (*p* = 0.4804). None of the queens in the Ovx-Rabies group had cytological signs of estrus/proestrus (i.e., >60% superficial cells) after ovariohysterectomy, while in the intact-Rabies group, two out of three queens showed such cytological signs. Interestingly, none of the Gonacon-vaccinated queens had signs of proestrus/estrus in the last 2 weeks of the study, excluding the non-responder queen in the GonaconX2-Rabies group. Furthermore, overall, among the cytology slides obtained from Gonacon-vaccinated cats, the percentage of superficial cells in the smear was significantly lower when the anti-GnRH titer was high (titer 0, 43.7 ± 8.7%, vs. titer 1:64, 10 ± 1.9%; *p* = 0.0135).

### Histology of the Reproductive Tract

**Figure 9:**
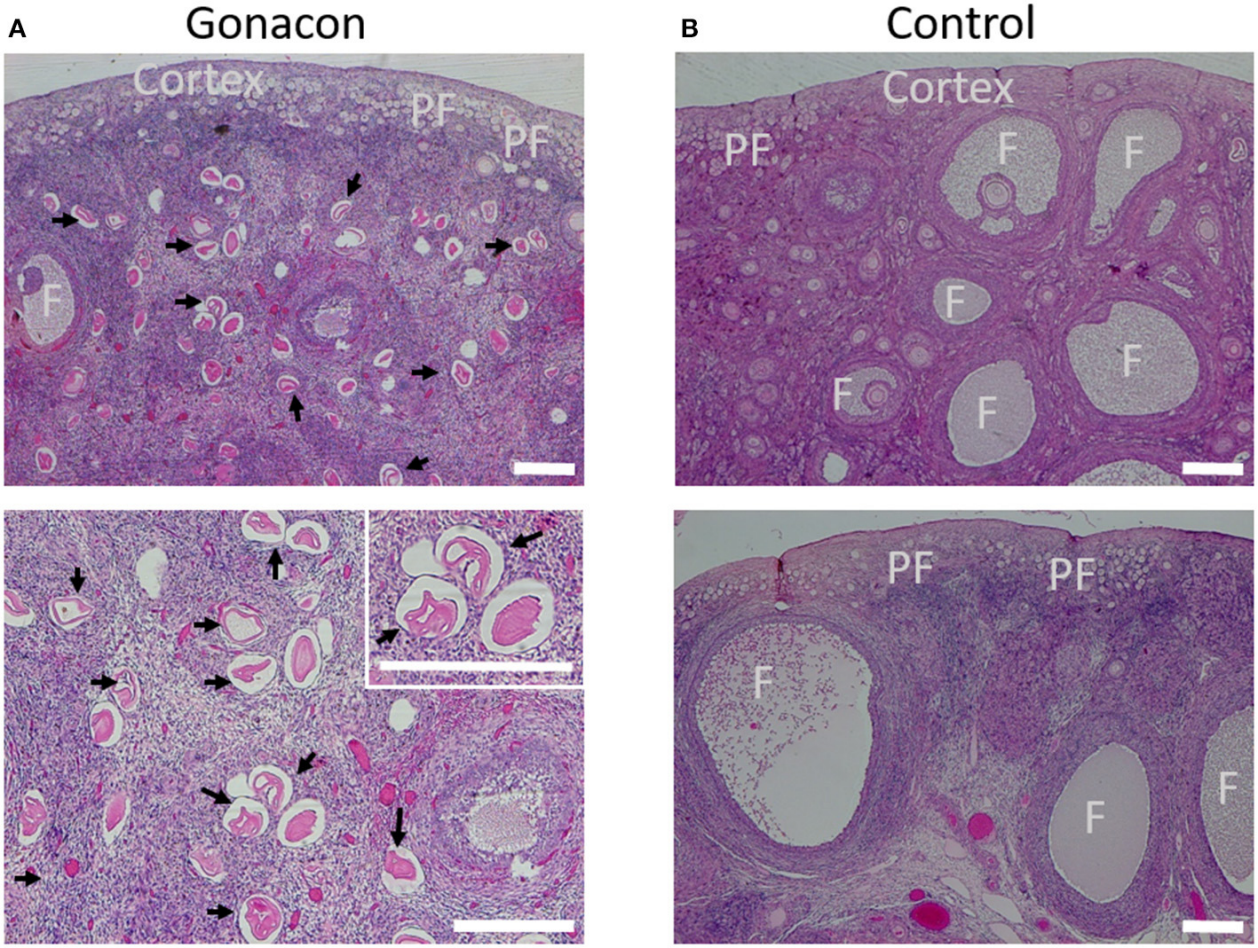
Histology of ovaries of Gonacon-vaccinated cats. Representative ovaries of Gonacon-vaccinated cats **(A)** are shown, as compared to ovaries of non-vaccinated cats **(B)**. Note the large number of degenerative oocytes (black arrows) in the ovaries of Gonacon-vaccinated cats. PF, crowded primordial follicles under the ovary's tunica albuginea, at the ovarian cortex; F, antral follicles. H&E staining. Scale bars in all images are 30 μm.

Histologic evaluation of the ovaries collected from Gonacon-vaccinated cats demonstrated many degenerative oocytes, and no to only a few very small antral follicles (Figure 9). In contrast, ovaries of cats that were not vaccinated with Gonacon had normal histology, with various follicles (primary, secondary, and antral follicle), and very few degenerative oocytes. The ovaries of the non-responder cat from the GonaconX2-Rabies were similar to that of non-Gonacon- vaccinated controls cats, with obvious large antral follicles, compatible with estrus. There were no apparent differences between Gonacon-vaccinated cats to controls in the shape, morphology, or amount of the crowded primordial follicles under the ovary's tunica albuginea, at the ovarian cortex. Furthermore, no obvious difference could be observed in the endometrium of Gonacon-vaccinated cats vs. controls (data not shown).

## Discussion

The rationale of this study was to examine the short-term safety and efficacy of combined vaccination with anti-GnRH (Gonacon) and rabies vaccines, specifically in female feral cats, which often suffer from disrupted health conditions and experience high-stress levels, which potentially may alter vaccination efficacy. Our study indicated that in the short term, the combined vaccination with Gonacon and rabies is safe and effective in female feral cats, with no apparent benefits for two Gonacon vaccinations as compared to a single dose. Over the study period, no local or systemic adverse health concerns were detected, in accordance with our hypothesis. There were no differences in serum rabies antibody titers among groups, and queens kept a protective titer throughout the study. Anti-GnRH antibodies were detected in all Gonacon-vaccinated queens, excluding one queen (GonaconX2-Rabies group). Furthermore, evaluation of serum AMH concentrations, vaginal cytology, and ovarian histology suggested that reproductive cyclicity was indeed suppressed in Gonacon-vaccinated queens, as hypothesized.

Different from previous studies in which researchers examined the effects of Gonacon in experimental cats or pets cats from colonies, in the current study we utilized feral cats. In previous Gonacon studies (30–33), animals were domestic short hair cats kept in excellent health and body conditions, and were accustomed to human interactions. In the studies of Levy et al. (30, 31), specific-pathogen-free (SPF) males and females, respectively, were acquired from a commercial vendor and housed in climate-controlled indoor spaces with controlled light cycles (30, 31). Vansandt et al. (32) used ovariohysterectomized cats from a research colony at the Cincinnati Zoo and Botanical Garden's Center for Conservation and Research of Endangered Wildlife. In the study conducted by Fischer et al. (33), well-maintained friendly cats were obtained from animal control agencies, or from private individuals who posted cats for rehoming on a classified advertisement website; all cats were in excellent body condition and health. However, it is well-documented that feral cats often suffer from high rates of pre-mature mortality and morbidity due to infectious and non-infectious diseases, internal and external parasites, starvation and poor body condition, weather extremes, trauma, etc. (9, 10, 34–36). Furthermore, most of these cats are not accustomed to contact with people and are typically too fearful and too wild to be handled. Therefore, feral cats often suffer from health issues and are under chronic stress conditions, which might weaken the immune response to vaccination (41), such as to the combined vaccination with Gonacon and rabies vaccines. Therefore, although some adjustments were needed in order to perform a study with feral cats as compared to well-maintained friendly cats (in regards to capturing, management, handling, cet.), the study was designed to examine the short term safety and efficacy of our combined vaccination approach specifically in feral queens, as they are the main target population for non-surgical contraception/sterilization.

Accordingly, in our study, the feral queens were captured by traps, and were maintained under ambient summer temperature and light. The study was conducted during the Israeli summer to avoid suppressing effects of the photoperiod or climate conditions on the reproductive system (8, 21, 53, 54). Furthermore, queens were vaccinated 24–72 h after they were captured, while their health condition was still typical to that of feral cats. Indeed, our physical examination and blood tests performed at study initiation, just prior to vaccination, indicated that many of the queens were in suboptimal body condition, and some had signs of anemia, inflammation, or muscle injuries. Furthermore, many cats had a high eosinophilic count in their blood, suggesting high infestation of gastrointestinal or other parasites. Despite the suboptimal health condition of the feral cats in the current study, the combined vaccination with Gonacon and rabies was safe and effective in the short term. None of the animals showed any local or systemic adverse reactions. In the study of Levy et al. (31), late-onset (2 years post-treatment) granulomatous injection-site masses developed in 5/15 (33%) of the Gonacon-vaccinated female cats. However, in another study conducted by the same group, no injection site masses were detected in male cats at a period of 6 months post-injection (30). In the study conducted by Vansandt et al. (32), four cats developed a sterile, painless, self-limiting mass at the site of Gonacon injection, after only few weeks. Fisher et al. (33) reported injection-site reactions ranging from swelling to transient granulomatous masses in 45% (*n* = 9/20) of vaccinated cats, beginning at least 1 month after treatment. Furthermore, in another study conducted in our laboratory (Novak and Raz, unpublished data), we also documented injection-site masses 3 to 12 months post-Gonacon vaccination in 7/44 (16%) owner-owned cats. Therefore, it is possible that in the current study, masses were not detected at all as the monitoring period of 14 weeks was relatively short; furthermore, we cannot rule out the possibility of local reactions later on, after the queens were released back to their environment or adopted. Regarding the systemic health conditions of the queens, although it was initially suboptimal for many of the animals, as typical for feral cats in Israel and other places, it improved over the study period. This improvement in the cats' health is probably due to the housing conditions during the study which allow the queens to recover; i.e., good availability of food, water, and shelter; reduced risks of infectious diseases and injuries; as well as reduced stress due to the acclimation of the cats to their environment.

Despite the initial suboptimal health condition of the feral cats in the current study, the combined vaccination with Gonacon and rabies was effective in the short term. Following vaccination with rabies, all queens from all the groups produced antibodies against rabies, which remained above the level considered protective. Bender et al. (29) vaccinated dogs with both Gonacon and a commercial rabies vaccine (Defensor 3, Pfizer, Inc., New York, NY, USA), and reported no adverse effects of simultaneous vaccination on rabies virus neutralizing antibody production. None of the previous studies measured rabies antibodies in Gonacon-vaccinated cats, even if cats were vaccinated with both vaccines (32, 33). Nevertheless, our results support our hypothesis that antibody titers against rabies will not be negatively influenced by the simultaneous vaccination approach and will remain protective during the study period.

Following vaccination with Gonacon, anti-GnRH antibodies titer increased in all vaccinated queens within 2–4 weeks, except for one non-responder queen from the GonaconX2-Rabies group. In all responder queens, anti-GnRH titer remained high during the study period, except for one queen from the GonaconX1-Rabies group, in which it reduced in the last two examinations. This queen was identified as FIV positive, which could have affected the immunogenicity of the Gonacon vaccine; however, the production of anti-rabies antibodies did not appear to be negatively affected in this queen. Nevertheless, the possibility of immunogenicity alteration of any immunocontraception vaccine due to FIV infection is interesting and warrants further investigation, as FIV might be quite prevalent among free-roaming cat populations (35, 55–57). There was no significant difference in GnRH antibodies between the GonaconX1-Rabies and GonaconX2-Rabies groups. However, as our study focused on the short-term effects, we cannot rule out the possibility that repeated Gonacon vaccinations would extend the effective period, and potentially provide extended contraception. Furthermore, as our assay was limited to series dilutions from 1:8,000 to 1:64,000, it is possible that the double Gonacon vaccination could have shown positive results in higher dilutions than 1:64,000. Previous studies showed that some cats did not respond to the Gonacon vaccine (i.e., no anti-GnRH antibodies), while others responded to the vaccine with anti-GnRH antibodies detected for various periods after injection. The only other study which compared a single to double Gonacon vaccination could not detect differences in the anti-GnRH antibody at 1:1,024 dilution at 4-month post-vaccination of ovariectomized queens (3 cats in each group) (32). Nevertheless, our hypothesis that two doses of Gonacon would induce higher anti-GnRH titer, as compared to a single vaccination, require further long-term study.

There are several relevant approaches to evaluate the function of the reproductive system following the administration of an immunocontraceptive agent, but it is clear that breeding provides the most definitive results. However, breeding trials, particularly in overpopulated species like cats, are practically challenging, require time and resources, as well as carry some ethical dilemmas, as to the destiny of the newborn offspring (or performing ovariohysterectomy of pregnant animals). Therefore, in the current study, we were looking for other alternatives. Accordingly, we evaluated the effects of Gonacon vaccination on the reproductive system by a combination of serum AMH concentrations measurements, vaginal cytology, and ovarian histology; which all suggested that the reproductive cyclicity was indeed suppressed in Gonacon-vaccinated queens. In females, AMH is a hormone produced in the ovaries by small and developing follicles, and has a pivotal role in the regulation of ovarian follicle reserve and folliculogenesis. Studies in other species have shown that there is a correlation between the number of developing follicles in the ovary and serum AMH concentration (58). It has also been found that when a queen is spayed, AMH serum concentration drops abruptly (50, 59). Accordingly, we initially hypothesized that AMH serum concentrations would be reduced following ovariohysterectomy, as well as following vaccination with Gonacon, due to the vaccine potential suppression effect on gonadotropins and folliculogenesis. Indeed, in the current study, AMH serum concentration was extremely low following ovariohysterectomies, but it remained high in intact queens, in agreement with previous studies in cats and other species (50, 59, 60). Interestingly, in queens vaccinated with Gonacon, the reduction in AMH concentration was gradual. Eventually, at study completion, AMH serum concentrations were lower in all Gonacon-vaccinated queens, as compared to study initiation, except in the non-responder queen from the GonaconX2-Rabies group, in which AMH concentrations remained stable throughout the study. Furthermore, statistical analysis of all serum samples obtained from Gonacon-vaccinated queen revealed that AMH concentrations were significantly lower in serum samples with high anti-GnRH titers (titer 0, 6.7 ± 1.1 ng/mL, vs. titer 1:64, 3.9 ± 0.7 ng/mL; *p* = 0.0429). Overall, these findings suggest, for the first time to the best of our knowledge, that measuring AMH serum concentration is a valuable method for evaluating the efficacy of immunocontraception.

Evaluation of vaginal cytology smears and histology of the ovaries at study completion indicated that none of the Gonacon-vaccinated queens was in estrus, except the non-responder queen. However, the assessment of these results, particularly those of the vaginal cytology, could have been clearer if other analyses could have done, such as measurements of other sexual hormones (e.g., estrogen, LH, and FSH), and ultrasonography of the reproductive tract. Nevertheless, the typical histological finding in the ovaries of Gonacon-vaccinated queens was the presence of a high number of degenerated oocytes, and lack of antral follicles, alongside a low percentage of superficial cells in vaginal smears. In contrast, in intact animals (samples of the Ovx-Rabies group just before spaying; and samples of Intact-Rabies at study completion), ovaries had normal histology, with various follicles (primary, secondary, and antral follicle), and very few degenerative oocytes. Interestingly, the ovaries of the non-responder queen from the GonaconX2-Rabies group were histologically similar to those of the controls, and the vaginal cytology smear had a high number of superficial cells (~90%) indicated that this female was in estrus. In our histologic evaluation, we were not able to detect apparent differences in the primordial follicle reserve in the ovaries, nor differences in the endometrium. Future studies should consider exploring the possible short and long-term effects of immunocontraceptive agents on the primordial follicle reserve, as the reduction or elimination of this pool would be beneficial for long-term contraception/sterilization.

Out of the three previous publications regarding Gonacon vaccination in female cats, two studies examined the contraceptive effect. In the long-term study conducted by Levy et al. (31), 15 queens were vaccinated with the early generation Gonacon vaccine, and were exposed to intact males. Gonacon-vaccinated queens re-gained fertility at a later time, as compared to controls; of the 15 vaccinated queens, 93% were infertile for at least 1 year, 73% for 2 years, 53% for 3 years, and 40% for 4 years; 27% were still infertile at the conclusion of the 5-year study. In the study conducted by Vansandt et al. (32), ovariectomized queens were used, and therefore, the possible effects of Gonacon vaccination on the reproductive system could not be determined. Later on, Fischer et al. (33) reported poor contraceptive efficacy of the Gonacon vaccine in colony cats. In their study, queens were exposed to intact males 4 months after vaccination with the newer Gonacon version. All control queens (*n* = 10/10) and 60% (*n* = 12/20) of Gonacon-vaccinated queens became pregnant within 4 months of the introduction of males. Two additional vaccinated queens became pregnant (70%; *n* = 14/20) within 1 year of treatment. Overall, vaccinated queens had a significantly longer (*P* = 0.0120) median time to conception (212 days), and lower fetal counts. Nevertheless, the results of their study were disappointing. In the current study, which utilized the same Gonacon formulation as in the study of Fischer et al. (33) but of different batch, our approach indicated that ovarian cyclicity was suppressed in all queens who respond to the Gonacon vaccine, as detailed above. The differences among studies could be due to variability in the vaccine itself [overtime differences in formulas, as reviewed by Benka et al. (15); different batches]; differences between cats (laboratory vs. free-roaming vs. stray cats); environmental conditions; as well as individual variability.

There are several limitations to our study. It is possible that due to the small number of cats in each of the groups, as well as the short duration of the study, we were not able to detect uncommon adverse reactions, or other safety issues, that could have been detected in a large group of vaccinated cats or at a later stage. Also, the validity of the vaccines' immunogenicity and the assessment of the proportion of non-responder cats (i.e., no antibody formation), as well as the assessment of reproductive effects, based on a small number of vaccinated cats examined over a short period, is limited. In regards to the assessment of the cats' health before and after vaccination, future studies should consider performing additional lab tests, such as urinalysis and fecal examination for the detection of intestinal parasites, which were not performed in our study, and could have provided a better understanding of the cat health status at vaccination. In regards to reproductive effects, other methods, such as assessment of other sexual hormones (e.g., estrogen, LH, FSH), ultrasonography of the reproductive tract, and breeding, should be considered and combined. In addition, during our study period, the queens were housed in individual cages, in conditions that were not similar to that of feral cats in an urban environment. Our study emphasizes the need to explore the safety and efficacy of the Gonacon vaccine in several target populations, and we therefore believe that further long-term studies are warranted, including in populations of stray cats in their natural environment, as well as in pet indoor vs. outdoor cats.

In summary, to the best of our knowledge, this is the first study that examined the short-term safety and efficacy of combined vaccination approach, which included anti-GnRH vaccine (Gonacon), given either as a single dose, or as two doses 3 weeks apart, in parallel with rabies vaccine, specifically in female feral cats. Overall, our results indicate that in the short term, the combined vaccination with Gonacon and rabies is safe and effective in female feral cats, with no apparent benefits for two Gonacon vaccinations as compared to a single dose. Following the combined vaccination, anti-GnRH and rabies antibodies were detected, despite the suboptimal health condition of the queens. Furthermore, evaluation of serum AMH concentrations, vaginal cytology, and ovarian histology suggested that reproductive cyclicity was indeed suppressed in Gonacon-vaccinated queens for at least 3 months. However, future studies should examine the long-term effects of contraceptive agents in free-roaming cats in their natural environment.

## Data Availability Statement

The original contributions presented in the study are included in the article/supplementary material, further inquiries can be directed to the corresponding author/s.

## Ethics Statement

The animal study was reviewed and approved by The Hebrew University's Institutional Animal Care and Use Committee.

## Author Contributions

SN: investigation, data analysis and interpretation, methodology, and writing—original draft. BY: conceptualization, investigation, data curation, writing—review, and editing. SS: investigation, data curation, writing—review, and editing. LM, ST, RN, and LJ: investigation, writing—review, and editing. RK: conceptualization, writing—review, and editing. DE: providing Gonacon vaccines, investigation, data analysis and interpretation, writing—review, and editing. TR: conceptualization, supervision, data curation, formal analysis, funding acquisition, project administration, investigation, methodology, validation, visualization, writing—review, and editing. All authors: contributed to the article and approved the submitted version.

## Conflict of Interest

The authors declare that the research was conducted in the absence of any commercial or financial relationships that could be construed as a potential conflict of interest.

## Abbreviations

AMH: Anti-müllerian hormone
LH: Luteinizing hormone
FSH: follicle-stimulating hormone
GnRH: Gonadotropin-releasing hormone
PCV: Packed cell volume
RBC: red blood cell count
MCV: mean corpuscular hemoglobin
MCH: mean corpuscular hemoglobin concentration
MCHC: mean corpuscular hemoglobin concentration
CHCM: cell hemoglobin concentration mean
CH: cellular hemoglobin
RDW: red cell distribution width
HDW: hemoglobin distribution width
WBC: white blood cells count
LUC: large unstained cells
ALT: alanine aminotransferase
GGT: gamma-glutamyl transpeptidase
AST: aspartate aminotransferase.
